# Survival of *Batrachochytrium dendrobatidis* in Water: Quarantine and Disease Control Implications

**DOI:** 10.3201/eid0908.030145

**Published:** 2003-08

**Authors:** Megan L. Johnson, Richard Speare

**Affiliations:** * James Cook University, Townsville, Australia

**Keywords:** *Batrachochytrium dendrobatidis*, chytridiomycosis, amphibian disease, research

## Abstract

Amphibian chytridiomycosis is an emerging infectious disease of amphibians thought to be moved between countries by trade in infected amphibians. The causative fungus, *Batrachochytrium dendrobatidis,* produces aquatic, motile zoospores; infections have been achieved in experiments by exposing amphibians to water containing zoospores. However, the ability of this fungus to survive in the environment in the absence of an amphibian host is unknown. We show that *B. dendrobatidis* will survive in tap water and in deionized water for 3 and 4 weeks, respectively. In lake water, infectivity was observed for 7 weeks after introduction. The knowledge that water can remain infective for up to 7 weeks is important for the formulation of disease control and quarantine strategies for the management of water that has been in contact with amphibians.

*Batrachochytrium dendrobatidis*, a member of the fungal order Chytridiales, causes amphibian chytridiomycosis, an emerging infectious disease (*1*). Amphibian chytridiomycosis has been responsible for massive illness and death in amphibian populations in Australia, New Zealand, Europe, United States, Central America, and South America (*2*–*7*). Amphibian chytridiomycosis in several continents has appeared in one region and subsequently spread as an epidemic wave. Data obtained by retrospective examination of museum specimens have identified the date of first occurrence as 1974 in the United States (*8*), 1978 in Australia (*9*), 1986 in Eduador (*10*), and 1999 in New Zealand (*7*). In 2001, amphibian chytridiomycosis was placed on the Office Internationale des Epizootes Wildlife Diseases List, the first time amphibian diseases had been listed.

*B. dendrobatidis* is an aquatic organism with two life stages: a sessile, reproductive zoosporangium and a motile, uniflagellated zoospore released from the zoosporangium. Frogs can be experimentally infected by zoospores of *B. dendrobatidis* (*11*,*12*) or by contact with skin harvested from infected animals (*2*). No resting stage has been identified for *B. dendrobatidis* in laboratory culture, and whether one occurs in nature is unknown as yet (*13*). The organism does not survive desiccation (*11*), so infected amphibians have been identified as the major means in which *B. dendrobatidis* could be moved within and between countries. Amphibians carrying *B. dendrobatidis* have been detected in the pet trade in the United States (*14*), Europe (*4*), and Australia (*15*); in frogs for scientific purposes, particularly *Xenopus laevis* and *X. tropicalis* (*16*–*18*); and in frogs for food, particularly *Rana catesbiana* (*19*). *B. dendrobatidis* has been hypothesized to have been introduced into new areas by movement of infected amphibians or in contaminated water or soil containing zoospores, but little is known about the epidemiology of amphibian chytridiomycosis (*15*,*20*,*21*).

Recent discussions have highlighted the need for research on the ability of this fungus to survive in the environment in the absence of a suitable host, since the fungus may be capable of a saprophytic life cycle (*13*). No data exist on the survival of *B. dendrobatidis* in water after an infected frog has been removed. We describe the survival of *B. dendrobatidis* introduced into autoclaved water from different sources. Autoclaved water was used since no currently available technique selects *B. dendrobatidis* from natural water bodies complete with bacteria, fungi, algae, and protozoa. We hypothesized that sterile water would represent the best possible opportunity for survival of *B. dendrobatidis* owing to the absence of competitor microorganisms.

## Methods

Water was collected from three sources in Townsville, North Queensland: deionized water, tap water collected from the chlorinated reticulated supply, and lake water from a 1-hectare lake in the suburb of Hyde Park. All water was then autoclaved at 121°C for 15 min. In duplicate 25-cm^2^ culture flasks (TPP, CSL Biosciences Ltd., Australia) containing each of the three water types, we added approximately 1 x 10^5^ spores/mL of 98-1810/3 of *B. dendrobatidis*, obtained from a wild adult of *Nyctimystes dayi* from Tully, Queensland, in 1998, or *B. dendrobatidis* strain 98-1469/10 isolated from a captive juvenile *Limnodynastes* *dumerilii* from the Amphibian Research Centre, Melbourne, Victoria, in 1998. Strains have been maintained by serial passage in TGhL broth (16 g tryptone, 4 g gelatin hydrolysate, 2 g lactose in 1,000 mL distilled water) approximately every week. Flasks containing water were held at 23°C in an incubator and observed during a 10-week period by using an inverted Olympus microscope (Olympus Optical Co., Ltd., Tokyo, Japan). Evidence of growth included attachment of inoculated zoospores, change in size and form of zoosporangia, and release of new zoospores into the water. Signs of viability included movement of free zoospores in the water or movement of zoospores inside the zoosporangia. From weeks 2 to 7, when activity of zoospores was no longer visible and growth appeared to have ceased, 0.5 mL of water from each flask was added into flasks containing TGhL media and incubated at 23°C. These newly inoculated cultures were then observed for growth and activity, with the observations terminating at week 10.

## Results

The duration of survival of *B. dendrobatidis* in water varied with the source of the water and the strain of fungus (Table). For all three water samples, zoospores attached to the plastic of the flasks and grew into zoosporangia, but new zoospores appeared to be released only into the lake water. After 1 week in tap and deionized water, no further growth of zoosporangia or activity by zoospores was apparent. However, viability of *B. dendrobatidis* was demonstrated in tap water until week 3 by growth of both strains on addition into culture media. For deionized water, viability of strains differed with growth on inoculation into culture media up to week 3 and week 4 for 98-1810/3 and 98-1469/10, respectively. The lake water contained dead microscopic algae, arthropods, protozoa, and plant debris, and zoosporangia were attached to these organic bodies as well as to the plastic of the flask (Figure). Growth of zoosporangia was apparent at week 1, and motile zoospores of both strains were present in lake water cultures for 1 to 7 weeks. However, during this period, no activity of zoospores was apparent at times. Subcultures from lake water into TGhL broth showed viability to week 6 and week 3 for the strains 98-1810/3 and 98-1469/10, respectively.

**Table:** Survival of *Batrachochytrium dendrobatidis* in artificial water environments

	Tap water	Deionised water	Lake water
Duration of growth in water^a^			
Strain 98-1810/3	1 wk	1 wk	1 wk
Strain 98-1469/10	1 wk	1 wk	1 wk
Release of zoospores			
Strain 98-1810/3	No	No	Yes (wk 5–7)
Strain 98-1469/10	No	No	Yes (wk 2,3,7)
Duration of viability^b^			
Strain 98-1810/3	3 wk	3 wk	6 wk
Strain 98-1469/10	3 wk	4 wk	3 wk

^a^Growth, zoospores attach to flask and develop into zoosporangia.

^b^Viability, growth occurs when aliquots from water are inoculated into TGhL broth.

**Figure:**
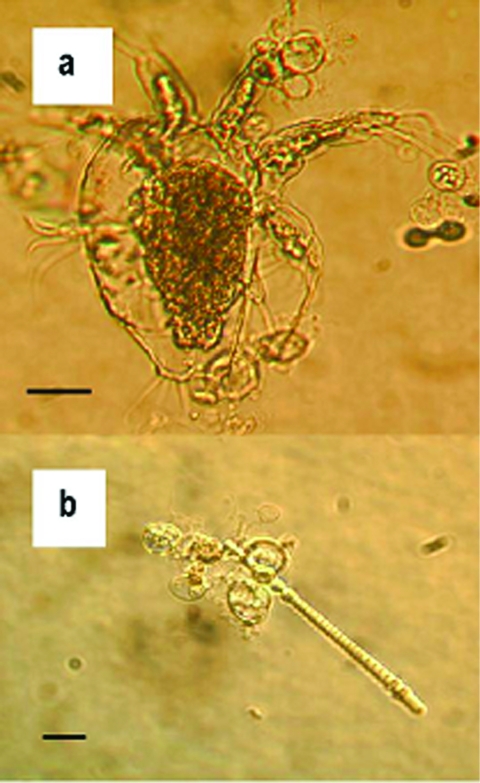
Zoosporangia of strain 98-1810/3 visible as transparent spherical bodies growing in lake water on (a) freshwater arthropod and (b) algae. Bars = 30 μM.

## Discussion

Zoospores of *B. dendrobatidis* were highly active when placed into all three types of water and progressively attached to the flask to form sessile zoosporangia. With routine culture in nutrient media, zoosporangia release zoospores into the medium mature as they mature. This cycle continues until the culture becomes overgrown and inhibition of growth occurs, possibly as nutrients become limiting or metabolic waste products accumulate. One cycle of normal growth for both these strains of *B. dendrobatidis* takes up to 5 days at optimum temperatures and nutrient conditions. However, in low nutrient conditions, such as exists in tap, lake, and distilled water, growth is inhibited and the life cycle takes longer to complete. To allow for a slower life cycle, observations were continued for 10 weeks. In our study zoospores developed to zoosporangia in all three water types, but new zoospores were not released in two of the three water types, tap water, and deionized water. Previous research had indicated that *B. dendrobatidis* did not survive well in distilled water (*11*). However, this study confirmed that the absence of activity by zoospores, or growth of new zoosporangia, is not a reliable indicator of a nonviable or dead culture. Zoosporangia may be able to survive in a state of arrested or nondiscernible development for a long period in environments not conducive to growth, and on inoculation into nutrient broth, the life cycle may then recommence. Results from inoculation of TGhL broth showed that *B. dendrobatidis* in tap water remained viable for 3 weeks. In deionized water strain 98-1810/3 survived for 3 weeks and strain 98-1469/10 survived 4 weeks. *B. dendrobatidis* in lake water was more successful: both strains were able to release new zoospores, or less likely, the original zoospores were capable of surviving for extended periods.

We presume that the longer survival time of *B. dendrobatidis* in lake water could be due to the higher level of nutrients and possibly the nonliving organic substrate offered by algae and other microorganisms. Attachment and growth of zoosporangia on skeletons of algae and invertebrates support this theory. However, no evidence of digestion of these bodies by the chytrid was visible. Repeated observations (Figure a) did not show any appearance of degradation. We do not know the effect of these microorganisms, if alive, on the growth and survival of *B. dendrobatidis*. Autoclaved water was used because of the limitations of our current culture system. Although active zoospores of both strains were observed in lake water cultures after 7 weeks, no growth occurred when subcultured into TGhL. Low numbers of zoospores were present. Therefore, when aliquots were removed to inoculate TGhL broth, zoospores may not have been included. Alternatively, zoospores may have been subject to osmotic shock when transferred from water to broth. *B. dendrobatidis* grows best when clusters of zoospores form (*13*), and too few may have survived inoculation to successfully establish a new culture.

These results have immediate relevance for disease control and quarantine strategies. Water in contact with amphibians should be regarded as contaminated with *B. dendrobatidis* for up to at least 7 weeks after last contact with the amphibian. For quarantine purposes, all water, moist soil, and wet fomites imported into a country with amphibians should be regarded as infectious for *B. dendrobatidis* unless the amphibians are shown to be uninfected. A similar strategy should be adopted when introducing new amphibians into a captive colony or collection. Similarly, water and any items coming into contact with amphibians moved within countries should be regarded as infectious for *B. dendrobatidis*. In practical terms, storage alone for a period of time should not be used as a means of ensuring water that has been in contact with an amphibian is not contagious. All water and wet soil in contact with an amphibian should be disinfected before discharge into the wastewater system or the natural environment. Amphibians should not be placed into enclosures with water used previously by other amphibians without prior disinfection. Any other wet objects that have been in contact with amphibians should either be disposed of or disinfected before repeat use. Various disinfection strategies have been described (M. L. Johnson et al, unpub. data). The most effective strategies for disinfection are heat (>47°C for 30 min), didecyl dimethyl ammonium chloride at >0.0012% final concentration for 2 min, or sodium hypochlorite (>1% for 1 min). To comply with the intentions of Office Internationale des Epizootes listing, amphibians, when moved between countries, should be placed in a different container on arrival; all water, soil, plants, and litter in contact with the amphibian during transport should be adequately disinfected by using techniques capable of killing *B. dendrobatidis*.
